# Bioengineering of *Bordetella pertussis* Adenylate Cyclase Toxin for Antigen-Delivery and Immunotherapy

**DOI:** 10.3390/toxins10070302

**Published:** 2018-07-20

**Authors:** Alexandre Chenal, Daniel Ladant

**Affiliations:** Institut Pasteur, Biochemistry of Macromolecular Interactions Unit, UMR CNRS 3528, Structural Biology and Chemistry Department, 28 rue du Docteur Roux, 75724 Paris CEDEX 15, France; alexandre.chenal@pasteur.fr

**Keywords:** adenylate cyclase toxin, antigen delivery, *Bordetella pertussis*, cancer immunotherapy, CD11b/CD18 integrin, cell-mediated immunity, cyclic AMP, dendritic cells, RTX repeats-in-toxin, toxin translocation

## Abstract

The adenylate cyclase toxin (CyaA) is one of the major virulence factors of *Bordetella pertussis*, the causative agent of whooping cough. CyaA is able to invade eukaryotic cells where, upon activation by endogenous calmodulin, it synthesizes massive amounts of cAMP that alters cellular physiology. The CyaA toxin is a 1706 residues-long bifunctional protein: the catalytic domain is located in the 400 amino-proximal residues, whereas the carboxy-terminal 1306 residues are implicated in toxin binding to the cellular receptor, the α_M_β_2_ (CD11b/CD18) integrin, and subsequently in the translocation of the catalytic domain across the cytoplasmic membrane of the target cells. Indeed, this protein is endowed with the unique capability of delivering its N-terminal catalytic domain directly across the plasma membrane of eukaryotic target cells. These properties have been exploited to engineer the CyaA toxin as a potent non-replicating vector able to deliver antigens into antigen presenting cells and elicit specific cell-mediated immune responses. Antigens of interest can be inserted into the CyaA protein to yield recombinant molecules that are targeted in vivo to dendritic cells, where the antigens are processed and presented by the major class I and class II histocompatibility complexes (MHC-I and II). CyaA turned out to be a remarkably effective and versatile vaccine vector capable of inducing all the components of the immune response (T-CD4, T-CD8, and antibody). In this chapter, we summarize the basic knowledge on the adenylate cyclase toxin and then describe the application of CyaA in vaccinology, including some recent results of clinical trials of immunotherapy using a recombinant CyaA vaccine.

## 1. Introduction

*Bordetella pertussis*, the causative agent of whooping cough, produces among a number of virulence factors an adenylate cyclase toxin, CyaA that plays a critical role in the early stages of respiratory tract colonization by the pathogenic bacteria [1,2,3]. CyaA is a 1706 amino acid long protein that is able to invade CD11b-expressing phagocytic cells, such as neutrophils and macrophages, where, upon activation by calmodulin (CaM), it produces supraphysiologic levels of cAMP and alters the phagocytic functions of these target cells [3,4,5]. CyaA has an original mechanism of invasion of target cells: after binding to the cell membrane it is able to deliver its N-terminal catalytic domain to the cytosol of target cells via a direct translocation across the plasma membrane. The original properties of CyaA, namely cell type selectivity and its unique pathway of entry into eukaryotic cells, have been harnessed to engineer recombinant CyaA proteins as a molecular Trojan horses able to deliver specific antigens into antigen presenting cells in order to elicit specific cell-mediated as well as humoral immune responses [6,7,8,9]. This chapter summarizes in a first part some basic knowledge about the adenylate cyclase toxin and in the second part the results of two decades of bioengineering of CyaA for antigen delivery and immunotherapy purposes.

## 2. *B. pertussis* Adenylate Cyclase Toxin, CyaA: Structure, Biogenesis and Mechanism of Action

### 2.1. CyaA: An Essential Virulence Factor of B. pertussis

The CyaA toxin is one of the major virulence factors of *B. pertussis* [1,3]. This toxin is secreted by virulent bacteria and is able to invade eukaryotic cells where it is activated by endogenous calmodulin to catalyze a massive production of cyclic AMP (cAMP) that results in profound alteration of cellular physiology.

The CyaA toxin plays a key role in the early stages of colonization of the respiratory tract by *B. pertussis* [10,11,12,13]. Pioneering studies from Weiss and collaborators showed that mutant strains that do not produce adenylate cyclase are avirulent in a mice model [10]. Confer and Eaton established in 1982 that partially purified preparations of adenylate cyclase were capable of inducing a large increase of intracellular cAMP levels in neutrophils and were concomitantly inhibiting the phagocytic functions of these cells [14]. Khelef et al. subsequently showed that *B. pertussis* causes apoptosis of alveolar macrophages ex vivo and that the CyaA toxin is the virulence factor responsible for this cytotoxic effect [15]. Later, Harvill et al. have shown, on a murine model of infection, that neutrophils and macrophages are, in vivo, the main targets of the CyaA toxin from *B. bronchiseptica*, a *Bordetella* species responsible for infection in animals [13]. Indeed, CyaA binds with high affinity and in a calcium-dependent manner, to CD11b/CD18 [16,17], an integrin—also called α_M_β_2_, Mac-1, or complement receptor 3, CR3—expressed by innate immune cells, which are therefore the main targets of the CyaA during infection [13]. The CyaA toxin thus appears to be a key mechanism of defense of the pathogen against the primary effectors of the innate immune response [1,3,4].

CyaA inhibits the phagocytic functions of neutrophils and macrophages by impairing chemotaxis and oxidative response, and eventually triggers cell apoptosis or necrosis [4,15,18,19,20,21,22]. This toxicity results mainly from a large increase in intracellular cAMP in target cells. Cyclic AMP is a major second messenger implicated in many biological processes. Accumulation of cAMP triggers a variety of cAMP-dependent signaling cascades that ultimately will paralyze phagocytic processes and profoundly affect gene transcription (particularly of many inflammatory genes). Besides, CyaA also exhibits a pore-forming activity that can alter membrane permeability [23,24,25,26,27]. In particular CyaA induces K^+^ efflux as well as Ca^2+^ influx [28,29]. These ionic imbalances also contribute to the overall cytotoxicity of the toxin [30,31]. In addition, CyaA may directly affect the adaptive immune responses by altering the maturation of dendritic cells [32,33] as well as the activation of T lymphocytes [34,35,36]. Besides, one should also keep in mind that during the course of infection, *B. pertussis* releases a variety of virulent factors that in addition to CyaA also contribute to the successful colonization of the host [2]. All these pathogenic components most likely operate in synergy although by large, the exact cooperative actions between the diverse virulence factors still remain to be unraveled [2].

### 2.2. Biogenesis and Structural Organization of CyaA

The adenylate cyclase is encoded by the cyaA gene [37] and its expression, like that of many other *B. pertussis* virulence genes, is controlled in a coordinated manner by environmental signals via a two-component regulatory system BvgS/BvgA [for a review, see [2]]. The CyaA toxin is synthesized as an inactive precursor of 1706 amino acids, proCyaA, that is converted into an active toxin, that is, able to invade eukaryotic target cells, by selective acylation of two lysine residues, Lys860 and Lys983 [38,39,40]. This co- or post-translational modification is carried out by a specific acyltransferase, CyaC, that catalyzes the transfer of an acyl group – mainly C16 - from acyl-ACP (Acyl-Carrier Protein) to the ε-amino group of the two lysines of proCyaA [39,40,41]. Sebo and colleagues showed that coexpression of CyaC and proCyaA in *Escherichia coli* is sufficient to obtain a fully functional cytotoxic CyaA toxin [42]. The acylated polypeptide is then secreted through the bacterial envelope by a type I secretion system (T1SS) that consists of three membrane proteins, CyaB, CyaD and CyaE [23]. The genes coding for these proteins are organized on the chromosome of *B. pertussis* in an operon with the cyaA structural gene [43]. The CyaB, CyaD and CyaE components assemble to form a continuous channel joining the inner and the outer membranes and the CyaA polypeptide is transported in an unfolded state through the channel of the T1SS secretion machinery directly from the cytosolic side to the external medium (for a review, [44,45]).

**Figure 1:**
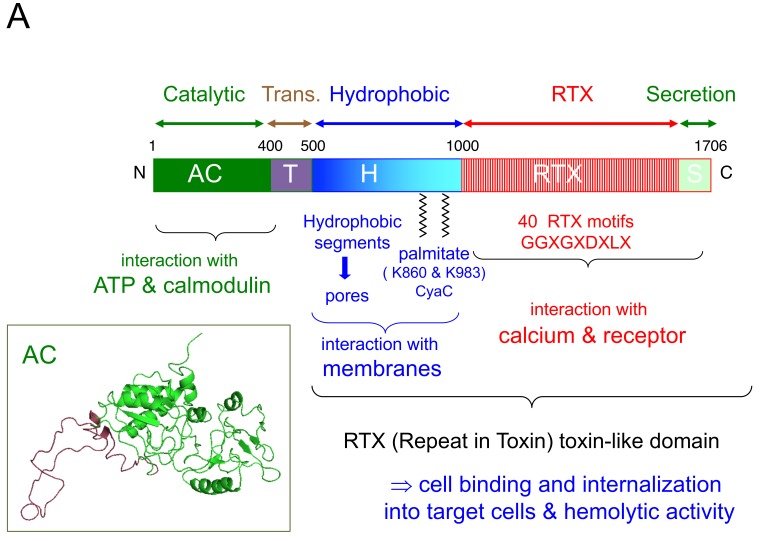

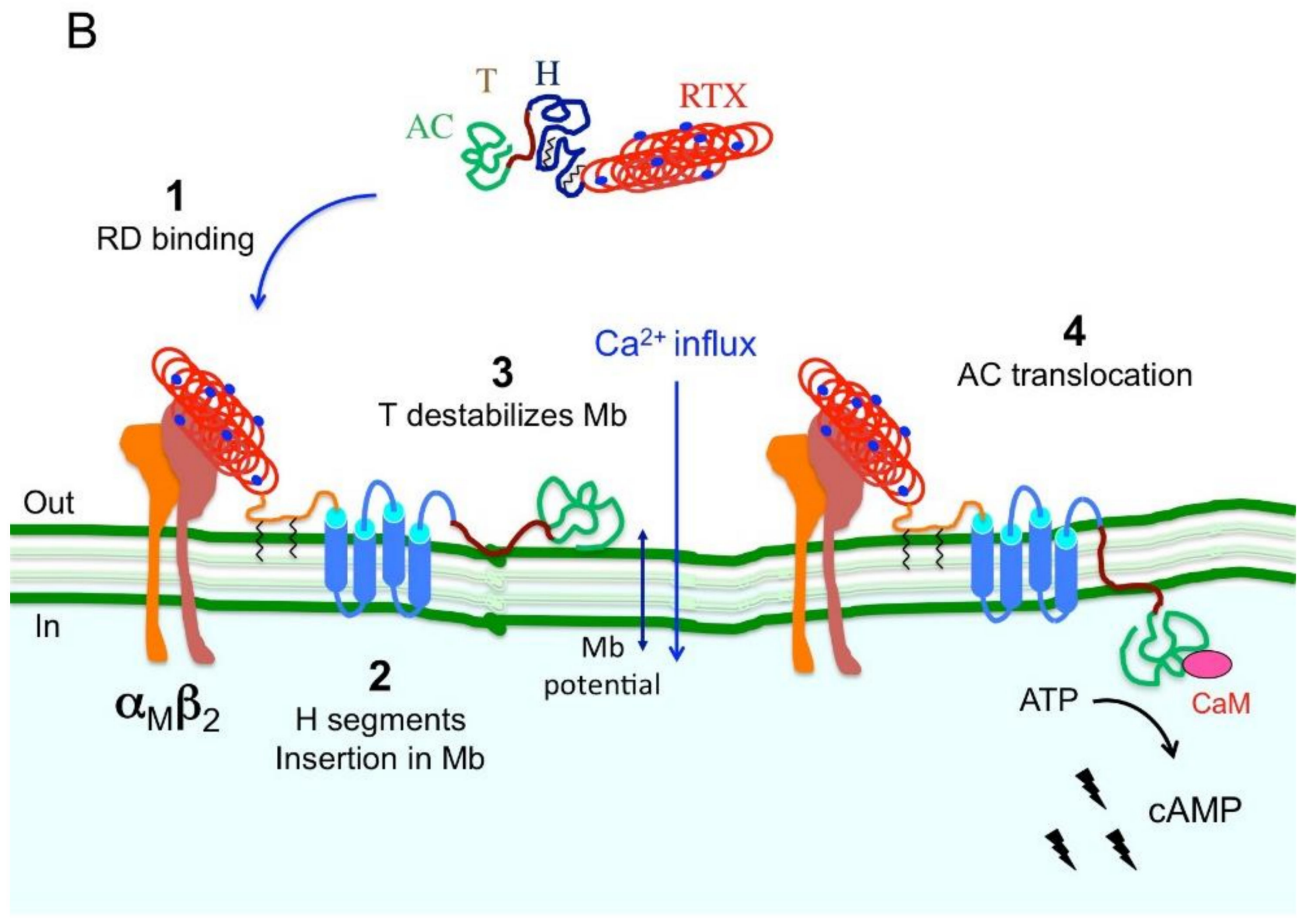
Structural organization of CyaA and schematic model of toxin entry into target cells. (**A**) The different domains of the protein are indicated. See text for details. (**B**) Model for CyaA intoxication of target cells: (1) binding of CyaA to CD11b/CD18 receptor at the target cell surface via its RTX domain; (2) insertion of hydrophobic segments of CyaA into the plasma membrane of target cells; (3) destabilization of the membrane by the translocation domain T; (4) translocation of the AC catalytic domain across the plasma membrane of target cells. In the cytosol CyaA interacts with calmodulin (CaM) that stimulates its catalytic activity to produce supraphysiological amounts of cAMP. This intoxication model and its different steps are still largely speculative, although it is reasonable to assume that binding of CyaA to its receptor (step 1) precedes membrane insertion of the hydrophobic domain (step 2). As discussed in the text, interaction of CyaA with CD11b/CD18 may primarily serve to recruit the toxin to the vicinity of the cell surface and thus facilitates the subsequent insertion of the hydrophobic helices into the membrane. In cells lacking the receptor, CyaA will bind and insert into the plasma membrane with a much lower efficiency. Once the hydrophobic domain is imbedded in the membrane, CyaA is irreversibly bound to the cells (irrespectively of the presence or not of CD11b/CD18) [16,24,38]. After insertion of the hydrophobic domain, the translocation domain (step 3) comes in close proximity to the membrane bilayer and triggers a local and transient destabilization of the membrane through its membrane active properties. The transient perturbation of the membrane bilayer integrity may favor the passage of the AC domain across the lipid bilayer (step 4) as well as a calcium influx that is associated with the entry of the AC domain [29]. Insertion of the hydrophobic domain (step 2) could also transiently destabilize the membrane to allow a K^+^ efflux and/or calcium entry [28,29].

CyaA is a bi-functional protein (Figure 1A) with an adenylate cyclase enzymatic activity and a hemolytic activity: the catalytic domain (responsible for the conversion of ATP to cAMP) is located in the 380 amino-terminal residues whereas the 1300 carboxy-terminal residues are responsible for the hemolytic activity, both activities being functionally separable [24,38,46,47,48].

The catalytic domain (AC) has a very high enzymatic activity (kcat = 2000 s ^−1^) when it is associated to its eukaryotic activator CaM [49], to which it binds with high affinity (KD < 0.1 nM) [50,51]. Guo et al. [52] solved the structure of the catalytic domain in complex with the C-terminal lobe of CaM and revealed how the CaM activator stabilizes the catalytic loops in a configuration favorable for high catalytic activity. Recent studies further explored the molecular basis of the allosteric activation of AC by CaM [53,54] and revealed that, in the absence of CaM, AC exhibits significant structural disorder [55]. CaM binding triggers a disorder-to-order transition of a central 75-residue-long AC segment (this segment is colored in red in the AC structure shown in Figure 1A) resulting in a long-range allosteric stabilization of the catalytic site [55]. The structural disorder of the AC domain may also be favorable for its translocation across the plasma membrane (see below).

The C-terminal part of CyaA (so-called hemolysin domain, residues 400 to 1706) has a low intrinsic hemolytic activity, which results from its ability to form cation-selective ion channels in biological membranes [26,47,56,57]. This domain is also essential for the binding of CyaA to the target cells as well as for the translocation of the catalytic domain through the plasma membrane of the target cells. Several distinct sub-regions (Figure 1) can be distinguished within the hemolysin domain:

- The region located immediately downstream to the catalytic domain (i.e., between residues 385–520) was shown to play a key role in the translocation of the AC domain across the plasma membrane of target cells. Within this “translocation region” (TR), the segment spanning residues 454–484 exhibits membrane-active properties. This TR region may be directly involved in a local destabilization of the lipid bilayer to favor AC translocation across the plasma membrane [58,59,60,61].
- A region between residues 520 to 720 contains several hydrophobic segments potentially able to form transmembrane α-helices. These segments are supposed to insert into the plasma membrane of the target cells and multimerize to form cation-selective pores [62]. Internal deletions and/or specific mutation within this region abolish or modulate both cytotoxicity and hemolytic activity of CyaA [23,24,56,62,63,64];
- A region that harbors the two lysines, Lys860 and Lys983, that are acylated by CyaC. The molecular mechanisms by which the addition of a fatty acid to these residues contributes to the toxicity of CyaA are not yet clarified although recent studies suggest that the acyl chains may have a structural role in favoring the folding of the CyaA into functional active states [65,66,67,68];
- A large C-terminal domain, the so-called RTX domain (residues 1000–1613) which comprises from about 40–50 copies of a repeating stretch of nine residues GGXGXDXLX (where X represents any amino acid). This nonapeptide sequence is a structural motif characteristic of a large family of bacterial toxins called RTX (Repeat in ToXin) toxins, the prototype of which is the α-hemolysin (HlyA) of *E. coli* [for reviews see [44,45,69]]. These RTX motifs constitute a new type of calcium binding structures that fold into a parallel helix-β-roll in the presence of calcium [70,71]. Calcium is indeed a key cofactor for CyaA as well as for all the RTX toxins [72,73,74,75]. CyaA binds about 40 calcium ions with submillimolar affinity [75,76]. Prior studies revealed that the RTX containing domain, RD, is natively disordered in the absence of calcium and acquired stable secondary and tertiary structures upon binding of calcium in a highly cooperative manner [77,78,79,80]. Hence, within the low calcium environment of the bacterial cytosol, the RTX motifs may adopt extended, intrinsically disordered conformations to foster protein export by the T1SS secretion machinery [71,80,81]. Upon reaching the extracellular environment enriched in calcium, these ions bind to the RTX motifs and trigger the folding of the whole toxin into its active conformation [67,68,71,80,82]. The calcium-induced disorder-to-order transition may thus be a key property for favoring secretion of CyaA as well as that of other RTX-containing toxins [45,81].
- The RD domain of CyaA has another essential function as it harbors the main binding site for the cell receptor, CD11b/CD18, located between residues 1150–1300 [16,17]. Importantly, the binding of CyaA to CD11b/CD18 is strictly calcium-dependent indicating that proper folding of the RD domain is required for this interaction.
- A secretion signal that is located at the very C-terminus of CyaA and that is recognized by the T1SS although the exact molecular features of this signal are not yet precisely defined [23,44,45,83].

### 2.3. Entry of CyaA into Target Eukaryotic Cells

The main originality of the CyaA toxin, with regard to other bacterial toxins, stems from its unique mechanism of entry into eukaryotic cells, which consists of a direct translocation of the catalytic domain across the cytoplasmic membrane of the cells [73]. Several experimental evidence supports this model of direct translocation:

- The process of intoxication (or internalization) is very rapid: an increase in cAMP in the target cells can be detected only few seconds after the addition of CyaA toxin; entry by endocytosis would require a much longer exposure time [72,73,84,85];
- The entry of CyaA is independent of the acidification of endocytic vesicles [86];
- Finally, CyaA can invade cells that have no membrane traffic, such as mammalian erythrocytes [72,73].

The molecular mechanisms by which CyaA penetrate the target cells are still largely elusive. This process can be schematized in several steps as described in Figure 1B:

The first step is the binding of CyaA to its receptor, the CD11b/CD18 integrin. CyaA can intoxicate many cell types, and it has been thought for many years that the toxin had no specific receptor. However, in 2001, Guermonprez et al. have established that CyaA binds with high affinity (K_D_ in the nM range) and in a calcium-dependent manner to the CD11b/CD18 integrin [16]. Indeed, the innate immune cells that express this receptor are the natural targets of CyaA during the infection (see above). 

The second step is the insertion of CyaA into the plasma membrane of the target cells. After binding of CyaA to the CD11b/CD18 receptor at the surface of the target cells, the hydrophobic segments (residues 520 to 720) of the protein may spontaneously insert into the plasma membrane. This anchoring, probably irreversible, preludes the translocation of the catalytic domain through the lipid membrane [16,17,87]. In the absence of CD11b/CD18, CyaA can also bind and insert into the membrane of the target cells, but with a lower efficiency and only at much higher toxin concentrations. [73,75,87,88,89]. Binding of CyaA to the CD11b/CD18 receptor thus permits the recruitment of the protein near the cell surface and thus facilitates its subsequent insertion into the membrane. In all cases, CyaA binding of the target cells, whether CD11b^+^ or not, requires the integrity of the C-terminal part of CyaA (i.e., residues 400 to 1706) as well as the acylation of the CyaA lysines K860 and/or K983 and is calcium-dependent [17,24,87,90]. Moreover, insertion of the toxin into the cell membrane triggers a potassium efflux as well as the formation of cation-specific pores that ultimately cause lysis of the cells. While the potassium efflux appears to be induced by monomeric form of CyaA inserted in the membrane [28], the pore-forming activity is clearly dependent upon the oligomerisation of the toxin [62].

The third step is the translocation of CyaA catalytic domain through the plasma membrane. The translocation of the CyaA catalytic domain into the cell cytosol is a process that depends on the temperature, the presence of calcium and the transmembrane potential of the target cells [27,38,72,73,74]. Several studies have revealed the importance of various residues and/or domains of the CyaA polypeptide in the translocation process (reviewed in [91]. It is well established that the catalytic domain can be delivered into the cytoplasm of the target cells directly through the plasma membrane in a process independent of endocytosis and vesicular trafficking [72,73,85,86]. Furthermore, Veneziano et al. demonstrated that CyaA can be translocated across tethered lipid bilayers. Using this synthetic model membrane, the AC translocation was found to be strictly dependent on the presence of calcium as well as on the membrane potential [92], as shown earlier in eukaryotic cells [27]. This suggests that the calcium-dependent CyaA translocation may be driven in part by the electrical field across the membrane. This artificial system thus indicates that CyaA can deliver its catalytic domain across a biological membrane without the need for any eukaryotic components (apart CaM that was used as a readout). However, the distinct steps followed by the CyaA polypeptide chain to pass across the membrane, remain uncharacterized at the molecular level. It has been proposed that the “translocation region” (TR) located downstream to the AC domain, and more particularly the peptide segment CyaA_454–484_ endowed with membrane-active properties, may contribute to a local destabilization of the lipid bilayer to favor passage of AC through the plasma membrane [58,59,60]. Furthermore, González-Bullón and colleagues have reported that CyaA has an intrinsic phospholipase A (PLA) activity that would determine AC translocation [93], but more recently, Bumba et al. reported contradicting results as they showed that highly purified CyaA is devoid of any detectable phospholipase A1 activity [94].

Another interesting issue is the potential role of the CD11b/CD18 receptor in the translocation process beyond the initial binding step. Indeed, Bumba et al. have shown that CyaA can mobilize its beta2 integrin receptor into lipid rafts where the effective translocation of AC domain across the target cell membrane would occur [95]. They showed that CyaA binding to CD11b/CD18 triggers an influx of calcium ions that activate calpain which then cleaves talin. This releases the integrin-CyaA complex that can diffuse to cholesterol-rich lipid microdomains where passage of the AC domain across the cell membrane would be favored. Whether the translocation is somehow distinct in CD11b^+^ and CD11b^−^ cells remains to be clarified. As said above, CyaA is able to invade a wide variety of cells that lack CD11b/CD18, and as a matter of fact, many studies of the CyaA translocation mechanism have been performed on CD11b/CD18-free cells, including mammalian erythrocytes [73]. With these cells lacking CD11b/CD18, while the overall binding of CyaA is low (only few percent of total CyaA), the translocation *per se* is very efficient as more than half of the bound CyaA molecules can deliver their AC domain into the target cell cytosol [73,88,96].

In addition to this direct intoxication pathway, CyaA can also be endocytosed especially in cells expressing the CD11b/CD18 receptor [97,98,99]. Finally, although the cytotoxicity of CyaA is mainly due to its ability to increase intracellular cAMP, the physiological consequences of the pore-forming activity of the toxin as well as its interaction with CD11b/CD18, a key signaling receptor leucocytes, remain to be precisely determined [3,100,101].

## 3. Applications of the CyaA Toxin as an Antigen Vaccine Vehicle

The *B. pertussis* CyaA toxin presents several properties that turned out to be particularly interesting for engineering an original non-replicating antigen-delivery vehicle. First, because of its ability to bind to the CD11b/CD18 integrin expressed on innate immune cells, CyaA can target in vivo a subpopulation of CD11b^+^ dendritic cells (DCs), which are professional antigen presenting cells (APCs) [16]. Second, CyaA can tolerate insertion of exogenous polypeptide fragments in its AC catalytic domain without impairing its ability to invade eukaryotic target cells [102]. Hence, antigens of interest can be genetically grafted into the catalytic domain of CyaA to generate recombinant toxins that can be efficiently targeted in vivo to CD11b^+^ DCs [103]. In these professional antigen-presenting cells, the grafted antigens are then processed and presented to both MHC-class I and class II pathways to induce specific CD8^+^ and CD4^+^ T cell responses respectively [7,8,9].

Results accumulated during the past two decades have established that CyaA is a highly effective and versatile vaccine vehicle, capable of inducing all the components of the immune response (T-CD4, T-CD8, and antibody). The immuno-therapeutic potential of this vector has been well demonstrated in various animal models and was also recently evaluated in human in clinical trials of cancer immunotherapy. We present below a brief summary of the main results and challenges in developing CyaA-based vaccines.

### 3.1. CyaA Can Deliver Antigenic Peptides into Antigen-Presenting Cells to Induce Specific T Cell Responses In Vivo

Initial studies of the structure/function relationships of CyaA permitted the identification in the AC catalytic domain of various “permissive sites” in which exogenous polypeptides could be inserted without significantly affecting the structure and the activity of the molecule [102]. As CyaA is able to deliver its AC domain through the cytoplasmic membrane of eukaryotic target cells, these results suggested that this toxin might be used as a vehicle to transport antigens of interest into the cytoplasm of antigen presenting cells (APCs) in order to activate specific cytotoxic T lymphocytes (CTL). These T cells, that express the cell surface glycoprotein CD8, recognize small peptidic fragments (so-called CD8^+^ T-cell epitopes) that are derived from the proteolytic degradation of the antigens, usually carried out by the proteasome in the cytosol of the cell, and that associate with the major histo-compatibility complex (MHC) class I molecules. Activated CTLs can lyse the cells that present the cognate MHC-I/CD8+ epitope complexes at their surface and therefore CTLs play a key role in the elimination of cells infected by viruses or intracellular pathogens, as well as in killing tumor cells that express specific tumor-associated antigens (TAA) [104,105]. Induction of efficient CD8^+^ T cell responses represents therefore a major goal for an effective vaccination against many intracellular pathogens and/or tumor cells. 

To explore whether CyaA could be engineered to transport CD8+ epitopes into the cytoplasm of antigen presenting cells (APCs), a sequence of 15 amino acids, corresponding to a CD8^+^ T-cell epitope of the lymphocytic choriomeningitis virus (LCMV) nucleoprotein, was inserted into various permissive sites of CyaA [106]. The recombinant proteins obtained retained their ability to invade eukaryotic cells and, moreover were able, in vitro, to sensitize the intoxicated cells to the cytolytic activity of cytotoxic T lymphocytes specific for the LCMV epitope. Hence, the LCMV epitope inserted in CyaA could be processed and presented to MHC-I molecules at the surface of the intoxicated cells [106].

These results were then extended to a variety of known CD8^+^ T-cell epitopes, and more importantly it was shown that mice immunized with these recombinant CyaA toxins develop strong, specific and persistent MHC I-restricted CD8^+^ CTL responses, independently of CD4^+^ T-cell help [107]. CTL induction required the full-length invasive activity of the recombinant CyaA toxin but interestingly, efficient CTL responses could be obtained with a genetically inactivated CyaA variant, CyaAE5, that lacks AC enzymatic activity as a result of a dipeptide insertion in the catalytic site and is therefore non-toxic. This established the proof of concept of using a genetically detoxified recombinant CyaA toxin (dCyaA) as a novel antigen vaccine vehicle for CTL activation.

Further experiments demonstrated that the antigen grafted in dCyaA follows the classical cytosolic pathway for MHC class I-restricted antigen presentation. Using the detoxified recombinant CyaAE5 protein carrying a T-CD8^+^ epitope derived from ovalbumin (OVA), Guermonprez and al. showed that the genetically inserted epitope in CyaAE5 is presented to CD8^+^ T-cells by a conventional mechanism that depends on proteasome activity, functional peptide-specific transporter TAP, and neosynthesis of MHC-I molecules [103,108].

As a first attempt to explore the potential application of dCyaA as a vaccine vector for immunotherapeutic purposes in human, Dadaglio and colleagues characterized recombinant dCyaAs harboring CD8^+^ epitopes derived from tumor-associated antigens (TAA) that are overexpressed in melanoma [104]. Two detoxified recombinant dCyaA proteins, each carrying a single copy of two human melanoma epitopes that associate with the HLA-A*02 haplotype, derived from tyrosinase or *N*-acetylglucosaminyltransferase V, respectively, were shown to induce potent epitope-specific CTL responses in transgenic mice expressing HLA-A*02 molecules [109]. In addition, in ex vivo assays, these recombinant proteins were efficiently presented by human dendritic cells to epitope-specific human cytolytic T lymphocytes isolated from melanoma patients [109,110]. From these results, the recombinant detoxified CyaA protein, dCyaA-Tyr, carrying the tyrosinase epitope (corresponding to residues 369–377 from the tyrosinase protein that is overexpressed in many melanoma) was chosen for further evaluation as an immunotherapeutic vaccine candidate in phase I clinical trials in melanoma patients.

**Figure 2:**
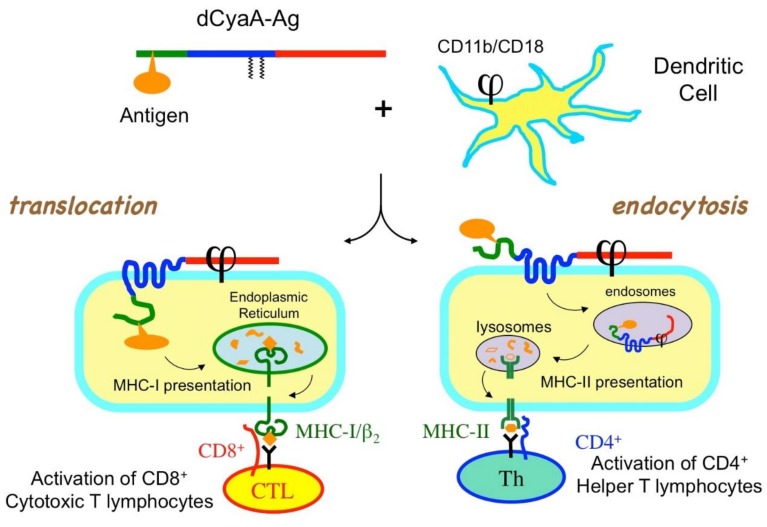
Recombinant CyaA proteins can deliver antigens into antigen presenting cells. Recombinant CyaA carrying an antigen genetically inserted into the catalytic domain, dCyaA-Ag, binds to dendritic cells (DC) as a result of its selective interaction with the CD11b/CD18 integrin receptor (ϕ). After translocation across the plasma membrane of the DC (**left**); the antigen is degraded by the proteasome into peptides that can reach the classical cytosolic MHC class I presentation pathway, to elicit specific CD8^+^ CTL responses. Alternatively, the recombinant CyaA can be endocytosed (**right**). After proteolysis in endosomes/lysosomes, the released peptides, including antigen-derived CD4^+^ epitopes, can enter the MHC class II presentation pathway, to elicit specific Th1 type CD4^+^ helper T-cell responses.

The selectivity of CyaA for dendritic cells in vivo could also be exploited to deliver MHC class II-associated epitopes and thereby stimulate CD4^+^ helper T-lymphocytes. Recombinant dCyaAs carrying CD4^+^ T-cell epitopes from the nucleoprotein of LCMV virus or the *E.coli* MalE protein were shown to induce potent specific CD4^+^ T-cell responses of Th1 type in the absence of any adjuvant [110,111]. The mechanisms of CyaA presentation of MHC class II epitopes were further analyzed by Schlecht et al. [112] using recombinant dCyaA carrying both class I (OVA) and class II (MalE) epitopes, as well as different inhibitors of intracellular trafficking. These authors showed that the antigens inserted into the catalytic domain of the toxin can be translocated into the cytoplasm of the APC to reach the class I epitope presentation pathway or alternatively be internalized by clathrin-mediated endocytosis to enter the endosome/lysosome class II epitope pathway (Figure 2). The ability of recombinant CyaA proteins to deliver antigens to both Class I and Class II MHCs is of particular interest given the importance of helper T lymphocytes in stimulating effective cytotoxic T-cell responses [8].

One interesting application of these recombinant CyaA proteins carrying *M. tuberculosis* epitopes is in the field of immuno-diagnostic, as illustrated by the work of Vordermeier et al. [113] and Wilkinson et al. [114] who showed that they could be used in ex vivo presentation assays to detect the presence of T cells specific for these mycobacterial antigens in infected patients or infected cattle.

Another synthetic design of CyaA vaccines was explored by Fayolle et al. [115] who described the grafting epitopes of interest into a recombinant CyaA vector by means of a chemical linkage through disulfide bonds. A recombinant dCyaA protein harboring a cysteine residue genetically introduced in the AC domain (CyaA has no native Cys residue) could be coupled in vitro with synthetic peptides corresponding to desired epitopes that were modified by appending at their N-terminus an additional cysteine residue activated with an nitro-pyridin-sulfonyl thiol group, Cys(NPys). These synthetic molecules elicited strong and specific CTL responses when injected to mice. This design represents a versatile method as a single CyaA carrier protein can be coupled to any desired synthetic peptide. Moreover it extends the range of applications of the CyaA vector as synthetic antigens containing lipidic or glycosidic determinants could also be grafted to the CyaA carrier.

### 3.2. Recombinant CyaA Proteins Induce Protective Immunity In Vivo

Saron et al. provided the first demonstration that a CyaA toxin carrying a viral CD8 ^+^ T epitope can stimulate effective protective immunity against viral infection [116]. Mice immunized with the CyaA-LCMV toxin (see above) developed strong CTL responses and were effectively protected when subsequently challenged intracerebrally with LCMV virus at doses that were lethal for non-immunized mice [116]. The observed protection was strictly dependent on CD8^+^ T-cells, but independent of CD4^+^ T-cells. Moreover, the protective immunity induced by the recombinant CyaA toxin was independent of the AC enzymatic activity [116]. CyaA could also confer significant protection against tumor proliferation. Two recombinant CyaA proteins (active or genetically detoxified) carrying the CD8^+^ OVA epitope (see above), induced strong epitope-specific CTL responses in mice against ovalbumin-expressing tumor cells [117]. Immunization of mice with these recombinant proteins significantly delayed the growth of ovalbumin-expressing tumor cells, thus prolonging the survival of immunized mice, as compared to control animals that received the wild-type CyaA protein or a placebo. Interestingly the detoxified CyaA vaccine appeared to be more protective against tumor growth than the enzymatically active variant. Moreover, the two recombinant CyaA proteins also confer effective therapeutic antitumor immunity that is, when tested in a setting where the CyaA vaccine proteins were administered after the tumor graft to the mice. Again a very significant protection was obtained, in particular with the detoxified variant [117].

In another model of infection, Tartz et al. showed that a detoxified CyaA protein carrying a CD8^+^ T cell epitope derived from the circumsporozoite protein (CSP) from *Plasmodium berghei* could protect mice from infection by this parasite, when used in a heterologous prime/boost vaccination protocol in conjunction with a live *Salmonella* vaccinal strain expressing the same CSP antigen [118].

### 3.3. Recombinant CyaA Proteins Induce Both Cellular and Humoral Immune Responses against Full Antigens

The initial experiments had been carried out mainly with recombinant CyaAs carrying short peptidic inserts but later studies then showed that CyaA can tolerate insertions of large polypeptidic fragments in its AC domain, and in several instances these large inserts (higher than 200 amino acid long) could be delivered into the cytoplasm of APCs [119]. Hence, not only small antigenic peptides but full-length antigens can be grafted into the CyaA vector. However, some constraints may arise from the electrostatic charge and/or the overall conformational stability of the grafted antigens which could restrict the general application of CyaA, in particular regarding the translocation of the antigens to the cytosol of DCs [96,119]. Thus appropriate engineering may be required to tailor specific antigens to the CyaA vector. 

A number of different recombinant CyaAs carrying various antigens from different pathogens have then been constructed and tested for their immunogenicity in mice model, as summarized below.

#### 3.3.1. Immunotherapy of Tumors Induced by Human Papillomaviruses

Infections by diverse human papillomaviruses (HPV) such as HPV16 and HPV18 are directly associated with the development of cervical cancer, the third most common cancer among women and the second most frequent cause of cancer-related death worldwide [120,121]. Two oncoproteins, HPV-E6 and HPV-E7, are expressed throughout the replicative cycle of the virus and are necessary for the induction and maintenance of transformation of infected cells. These proteins are therefore considered to be attractive tumor-associated antigens and good targets for immunotherapy [122]. Preville et al. [123] produced recombinant detoxified CyaAs carrying either the entire HPV16-E7 protein (98 amino acids) or a variety of sub-fragments. These recombinant proteins when administered to mice in the absence of any adjuvant were capable of inducing strong E7 antigen specific CTL responses as well as Th1 type CD4^+^ responses, and could completely eradicate pre-established murine tumors expressing the E7 antigen [123]. This remarkable therapeutic efficacy was obtained after a single administration of 10 μg (about 50 pmol) of the recombinant CyaAs by the subcutaneous route and in the absence of any adjuvant. These molecules could thus constitute a simple and effective immunotherapeutic approach against HPV-induced tumors. Clinical trials to evaluate the efficacy of this therapeutic vaccine in human have been carried out by the Genticel company (now Genkyotex) as described below in Section 3.5.

#### 3.3.2. Mycobacterium Tuberculosis

Recombinant CyaAs carrying major immunogenic determinants from *Mycobacterium tuberculosis*, i.e., the immunodominant regions of antigen 85A, the complete sequence of the 6-kDa early secreted antigenic target (ESAT-6) proteins or the TB10.4 antigen, were shown to induce potent antigen specific Th1-polarized T-cell responses in mice [124,125]. Animals immunized with CyaA-85A or CyaA–ESAT-6 in the absence of any adjuvant developed strong antigen-specific lymphoproliferative, interleukin-2 (IL-2) and gamma interferon (IFN-γ) cytokine responses, in the absence of any IL-4 or IL-5 production. Furthermore, these proteins were able to markedly increase the intensity (and frequency) of Th1-polarized CD4^+^ T cell responses when administered as booster after initial priming with a BCG vaccinal strain expressing ESAT-6, although these enhanced responses did not translate into better protection of mice against *M. tuberculosis* infection [124].

#### 3.3.3. Human Immunodeficiency Virus (HIV)

The transactivator of transcription Tat protein (86–101 amino acids, depending on HIV subtype) is a key virulence factor from the human immunodeficiency virus (HIV) and is considered as an optimal candidate for therapeutic immunization against HIV [126,127]. Mascarell et al. [128] produced a recombinant detoxified CyaA protein, dCyaA-Tat carrying the whole Tat antigen, an 86 residue long polypeptide, inserted between residues 224 and 225 of CyaA and tested its capacity to induce HIV-Tat-specific cellular as well as humoral immunes responses. The dCyaA-Tat protein when administered to mice in the absence of adjuvant elicited strong Th1 polarized responses and primed Tat specific IFN-γ producing CD8^+^ T-cells. The dCyaA-Tat vaccine also induced strong and long-lasting anti-Tat antibody responses that have the ability to neutralize the transactivating function of the native Tat protein in vitro [128]. In another study, Fayolle and colleagues characterized recombinant dCyaAs harboring the Tat antigen grafted at various position in the toxin and/or carrying multiple copies of the dominant Tat: 1–21 B-cell epitope : these latter proteins were shown to induce high titers of anti-Tat antibodies, even after a single immunization, that persisted up to 10 weeks post-immunization [129].

The immunogenicity of CyaA-Tat vaccine in non-human primates was examined by Mascarell et al. [130]. African green monkeys immunized with dCyaA-Tat either in the presence or in the absence of alum adjuvant, developed strong anti-Tat antibody responses that could bind to Tat and neutralize its transactivating function in vitro. When administered in the absence of alum, CyaA-Tat also induced Th1 Tat specific T cell responses. Hence the CyaA vector is efficiently delivered to DCs in non-human primates and is capable of eliciting all components of the immune response (CD4^+^ and CD8^+^ T-cells and antibodies).

Another important aspect is the observation that prior immunity against the CyaA protein does not significantly interfere with the induction capabilities of cellular immune responses by recombinant CyaA proteins carrying a heterologous antigen as illustrated in various studies [110,123,130,131].

### 3.4. Recombinant CyaA Vaccines Target Dendritic Cells In Vivo and Induce Their Maturation 

One of the key properties that explains the high immunogenic potency of CyaA vector derives from the capacity of this protein to target in vivo a subpopulation of dendritic cells that express its cellular receptor, the CD11b/CD18 integrin (or α_M_β_2_). Indeed, Guermonprez et al. [132] demonstrated that in vivo, CyaA is selectively targeted to CD11b^+^ “myeloid” DCs. Following intravenous administration of the detoxified CyaA-OVA recombinant protein (i.e., carrying the OVA epitope, see above), the MHC-I/OVA complexes were mainly displayed by the CD11c^+^ CD8^+^ CD11b^high^ dendritic cells. Selective antigen targeting by CyaA on myeloid dendritic cells is sufficiently effective to induce CTL responses in the absence of any adjuvant and/or CD4^+^ T helper lymphocytes [132].

The remarkable efficiency of the CyaA vaccines to stimulate the various components of immune responses—i.e., CD8^+^ cytolytic T-cells, CD4^+^ T helper lymphocytes and antibody responses—has long suggested that this vector might be endowed with a specific capacity to trigger maturation of the DCs. Effects of wild-type CyaA on innate immunes cells, macrophages and neutrophils or DCs, have been well documented (reviewed in [3,133]) but these activities are primarily dependent upon the capacity of CyaA to rise cAMP in intoxicated cells. As the CyaA vaccines are usually detoxified variants (i.e., without AC enzymatic activity), these cAMP-dependent signaling processes cannot be involved.

An additional issue in all these studies is that CyaA as it is purified from Gram-negative bacteria (either the native toxin from *B. pertussis* or as a recombinant protein expressed in *E. coli*), is potentially contaminated by other proinflammatory bacterial contaminants such as lipopolysaccharide (LPS), flagellin, peptidoglycan fragments, etc. As a matter of fact, most of studies carried out until 2003–2005 were done with CyaA preparations massively contaminated by LPS. Introduction of specific cleaning steps (as described in Preville et al. [123]) permitted to reduce LPS contaminations to acceptable levels for vaccine injection. Yet, presence of residual LPS precluded any sound analysis of the putative immunomodulatory properties of the detoxified CyaA. Only recently Dadaglio and colleagues [134] could reasonably examine this critical issue, by taking advantage of the availability of a highly purified recombinant CyaA protein, dCyaA-Tyr, that had been produced under Good Manufacturing Practice (GMP) conditions for a Phase I clinical trial.

These authors showed that this highly pure, detoxified CyaA vaccine that contains extremely low levels of LPS (less than 10 EU/mg), can bind to CD11b^+^ dendritic cells (DCs) and induce their maturation—as revealed by the upregulation of costimulatory and MHC molecules and the production of proinflammatory cytokines - via a signalling process involving the TLR4/TRIF pathway [134]. Hence the recombinant detoxified CyaA proteins not only target DCs in vivo but are also able to activate these cells [134]. Yet, the precise molecular mechanism of TLR4 activation by detoxified CyaA still remains to be clarified. Binding of dCyaA to CD11b/CD18 integrin may favor a TLR4/CD11b interaction, or dCyaA could directly interact with TLR4 via its acyl chains. Alternatively, the pore-forming and K^+^ efflux activities of dCyaA may also contribute to the maturation of CD11b-expressing dendritic cells (DC) as proposed by Svedova et al. [135]. Dunne et al. had shown earlier that CyaA could induce robust IL-1beta production by dendritic cells through activation of caspase-1 and the NALP3-containing inflammasome complex, a process that was dependent on the pore-forming capacity of CyaA, but not on the AC enzymatic activity [136].

Whatever the exact mechanism involved, the ability of detoxified CyaA proteins to trigger the maturation of CD11b^+^ DCs is certainly a key property that may explain the high efficiency of the recombinant CyaA vector to induce strong immune and polyvalent responses in vivo. This property may also be implicated in the adjuvanticity and immunomodulatory capacities of native and detoxified CyaA toxin as reported by different authors [137,138,139] who showed that CyaA, when co-administered with various antigens, can enhance the serum IgG antibody responses to each of those antigens.

### 3.5. Clinical Trials of CyaA Vaccine in Immunotherapy of Human Papillomavirus Induced Tumors

The above-described results establish that the dCyaA protein is a highly effective non-replicative vaccine vector for delivering antigens in dendritic cells and stimulate specific, cell-mediated and humoral immune responses. Yet, the “proof of concept” of CyaA as a vaccine vector was essentially obtained in murine models. Clinical trials to evaluate the vaccine potential of CyaA in human were initiated by the biotech company Genticel (now Genkyotex) focusing on the immunotherapy of tumors induced by human papillomaviruses. As described above, recombinant dCyaAs carrying the HPV16-E7 antigen were able to elicit, in mice, potent CTL as well as Th1 CD4^+^ responses that could efficiently eradicate pre-established E7-expressing murine tumors, TC-1 [123,140].

A phase I clinical trial (EudraCT No. 2010-018629-21) was carried out with a bivalent vaccine made of two different detoxified CyaAs carrying the E7 antigen from HPV-16 and HPV-18 (the two most prevalent subtypes associated with cervical cancer), that were produced separately in GMP conditions and combined in equal amount in a single vaccine (GTL001). GTL001, when administered intradermally in mice with a TLR3 agonist (polyinosinic-polycytidylic acid) or a TLR7 agonist (topical 5% imiquimod cream) adjuvant, induced strong HPV16 E7-specific T-cell responses capable of eradicating HPV16 E7-expressing tumors. Antigen-specific memory T-cell responses were detected in tumor-free mice that were thus protected against a subsequent challenge with HPV18 E7-expressing tumor cells [131,141].

A phase I clinical trial (EudraCT No. 2010-018629-21) enrolled 47 women infected with HPV16 or HPV18 but with normal cytology (e.g., no or low grade lesions), with a primary objective to test the tolerability and immunogenicity of GTL001 [142]. The GTL001 vaccine (100 μg, low dose, or 600 μg, high dose) or placebo were administered by intradermal injections (2 vaccinations 6 weeks apart) and adjuvanted (or not) by topical application (15 min and 24 h after each injection at each injection site) of a 5% imiquimod cream (Aldara^TM^) or a placebo cream in control groups. Imiquimod is a synthetic imidazoquinolone that activates NF-kB (*via* TLR 7 and TLR 8) and induces pro-inflammatory cytokines and chemokines. The results showed that GTL001 adjuvanted with topical imiquimod cream had an acceptable safety profile (no toxicity and only transient and mostly mild local reactions) and could induce antigen-specific cellular immune responses. Virology results also suggested that GTL001/imiquimod might enhance HPV clearance, although this phase I trial was not designed to detect significant differences in viral clearance [142]. These results were thus interesting enough to support a larger phase II study to evaluate the vaccine efficacy.

The Phase II clinical trial (RHEIA-VAC) was a randomized, placebo-controlled, double-blind trial carried out at 39 sites in 7 countries. 233 HPV16/18 positive patients at baseline with normal (NILM1) or abnormal (ASCUS and LSIL1) cytology were enrolled (one arm of 117 patients for treatment and one arm of 116 patients for placebo). They received two intradermal injections of 600 μg GTL001 or placebo, 6 weeks apart, followed by 2 applications of 5% imiquimod cream, 15 min and 24 h after each injection. As primary endpoint, patients in both groups (vaccine or placebo) were assessed for viral clearance (with a quantitative HPV PCR genotyping assay) after 24 months. However, the results, released in December 2016 by Genticel, revealed no significant difference in viral clearance between the treatment group and the placebo group during 2 years of treatment as well as no difference between the groups in terms of incidence of progression to high grade cervical lesions. Genticel then announced stopping the GTL001 vaccine development program and merged with Genkyotex (www.genkyotex.com) to focus on finding new drug candidates.

## 4. Conclusions and Perspectives

The absence of noticeable therapeutic effects of the recombinant CyaA-based vaccine GTL001 in Phase II trial in humans was obviously quite disappointing for the field. Yet it should be tempered by several considerations:

- Cancer immunotherapy remains a daunting challenge. Many different trials are currently focusing on the treatment of HPV-induced premalignant or cancerous lesions [121] with limited success thus far. One key issue is the local immune suppression that may impede the efficacy of therapeutic vaccines. Different approaches to dampen the general immune suppression are being explored in various trials [121] and would certainly improve as well the potency of CyaA-based vaccines.
- Berraondo et al. [140] indeed previously demonstrated that very large E7-expressing murine tumors (TC-1) could be efficiently eradicated upon administration of the dCyaA-HPV-E7 vaccine in combination with TLR ligands (e.g., CpGs that target TLR9) to stimulate dendritic cell maturation in vivo, and immunosuppressive molecules, such as cyclophosphamide that, at low doses, can suppress regulatory T cells (Tregs) to allow a more effective induction of antitumor immune responses [140]. Thus, more effective therapeutic intervention may benefit from such combinatorial strategies that simultaneously target the innate, adaptive, and regulatory components of the immune system.

Another main possible direction for the enhancement of the CyaA-vaccine efficacy would be to enlarge the diversity of targeted antigens. An obvious candidate for HPV therapeutic vaccines is the E6 oncoprotein, which is a major tumor associated antigen in HPV transformed cells. Indeed, many of the ongoing therapeutic HPV vaccines are simultaneously targeting both the E6 and E7 antigens [121]. It would be worth to test a combination of two CyaA vaccines harboring either E6 or E7 to increase the polyvalency of the immune responses as well as combining them with other vaccine approaches that might synergize to provide an enhanced immunogenicity.

Besides, the CyaA vector itself may also be rationally implemented to increase the efficacy of antigen delivery and processing. Better biochemical and physicochemical characterizations of the recombinant CyaA vaccines may help to improve the formulation and stability of the CyaA immunogens. From a fundamental point of view, a deeper understanding of the CyaA mechanisms of action, and particularly of its translocation process across the plasma membrane of target cells is critical. How the insertion of exogenous antigens may affect the structure, folding, stability and/or function of the recombinant CyaAs should also be clarified. As a matter of fact, very few is known of the precise processing of the antigens delivered by the CyaA vector inside the APCs. Whether or not the overall efficacy of antigen presentation by the MHC class I pathway might be manipulated in order to enhance the overall immunogenicity of the CyaA vaccines, remains to be explored. Finally, it might be possible to engineer the detoxified CyaA in order to enhance its capacity to activate DCs. Addressing all these questions may ultimately help to design improved CyaA vectors on a more rational basis.
